# Prevention and mitigation strategies for psychological disorders in European ongoing and post-war settings: lessons from the 2022 Russo-Ukrainian war

**DOI:** 10.3389/fpsyg.2026.1934406

**Published:** 2026-09-09

**Authors:** Seth Davin Norrholm, Michelle Szewczuk, Tanja Jovanovic

**Affiliations:** Department of Psychiatry and Behavioral Neurosciences, Neuroscience Center for Anxiety, Stress, and Trauma, Wayne State University School of Medicine, Detroit, MI, United States

**Keywords:** coping, migration, posttraumatic stress disorder, trauma, war

## Abstract

There is an ongoing need to facilitate the development and implementation of preventative measures and prophylactic steps to mitigate the development of psychiatric disorders such as posttraumatic stress disorder (PTSD) within traumatized populations in theaters of war (e.g., 2022 Russo-Ukrainian War). If successful, these measures have the potential to reduce the incidence, severity, and duration of psychological distress and posttraumatic stress reactions, including PTSD, while increasing the likelihood of resilience. This conceptual analysis paper will review the psychological effects of either living in, or being displaced from, active war zones in Europe. The ongoing situation in Ukraine will be used as an example, with specific emphasis on risk and resilience factors, prevention strategies spanning therapeutic domains, and how we can apply what the medical and scientific communities have learned in the aftermath of other global armed conflicts historically. As such, the paper will close with a discussion of the empirically recommended mental health interventions and tools that should be made available to the non-displaced Ukrainian population, internally and external displaced individuals (refugees), as well as citizens of host countries that receive refugee populations.

## 1 Introduction: 2022 Russo-Ukrainian war-related trauma exposure

Mental health disorders are a rising global concern with recent estimates by the Global Burden of Disease Mental Disorder Collaborators showing that 1.17 billion people were living with these disorders in 2023 which represents twice the prevalence reported in 1990 (GBDMDC, 2026). In the latter study, anxiety-related disorders, including posttraumatic stress disorder (PTSD), represented one of the leading global mental health disorders of concern (11th out of over 300 reported diseases and injuries). To date, the posttraumatic effects of civilians either living in or displaced from war-torn regions has largely focused on countries designated as High Income Countries (HICs) by the World Bank with little to no investigation of these psychological effects in designated Middle- or Lower Income Countries (MLICs). This is especially important given that the lower income countries populations are at greater risk for developing posttraumatic stress reactions (de Jong et al., 2001). Ukraine represents an MLIC at greater risk for mental disorders with one in three Ukrainian citizens reporting symptoms or problems that meet diagnostic requirements for a mental health disorder (Martsenkovskyi et al., 2024), most notably, following the 2014 and 2022 Russian hostilities directed at this European country.

With regard to war-related trauma exposure, Ukrainian citizens first experienced the initial annexation of Crimea by Russia in 2014 and then the four years that have elapsed since Russia invaded Ukraine on February 24, 2022. These Russian military operations have led to numerous types of DSM-5 Criterion A trauma (APA, 2013) for the Ukrainian population including, but not limited to, genocide, bombing and artillery fire, targeted shelling of civilian and residential buildings, learning about a friend or loved one that was physically hurt or killed, torture, destruction of infrastructure, and sexual assault of women, men, and children (Borger, 2022; Beaumont, 2022; Chao-Fong et al., 2022; Mckernan, 2022). As of July 2026, the Ukrainian population experienced 16,402 civilian deaths and 548,428 injuries since the February 2022 invasion (although this number is the subject of some debate among countries and agencies) (UN, 2026). Estimates of posttraumatic stress disorder (PTSD) prevalence in Ukraine have been reported to be between 26 and 73% (Lushchak et al., 2024; Chudzicka-Czupala et al., 2023; Karatzias et al., 2023) of the general population and these estimates include both internally displaced individuals (IDPs) and refugees who left for other countries. Not surprisingly, the most significant predictor of PTSD (as well as anxiety and depression) in Ukrainians has been a high degree of exposure to the aforementioned war-related stressors (Hamama-Raz et al., 2022; Karatzias et al., 2023; Kurapov et al., 2023; Kurapov et al., 2022) to the extent that a dose–response association has been observed between the number of traumatic events experienced and one’s PTSD severity (Wang et al., 2024; Hyland et al., 2023; Chudzicka-Czupala et al., 2023).

## 2 Effects on the Ukrainian population in general

As discussed by Johnson et al. (2022) and several other groups, there are leading biological, experiential, social, and cultural factors that may affect risk versus resilience for developing posttraumatic reactions in the Ukrainian population. Significant *risk* factors include, but are not limited to, frequency of trauma exposure (e.g., number of DSM-5 criterion A events), PTSD symptom severity (e.g., frequency, cluster type, and intensity of symptoms), age at the time of trauma exposure, strength of ethnic ties to the war-torn country (i.e., patriotism), proximity to war-related violence, unavailability of resources that promote resilience and survival, financial instability, loneliness, being a female, experiences of discrimination, cognitive impairment, disturbed sleep, and peritraumatic dissociation (Cyniak-Cieciura et al., 2022; Brown et al., 2018; Fox et al., 2021; Hawkley and Cacioppo, 2010; Bilewicz et al., 2024; Hamama-Raz et al., 2022; Avery et al., 2023; Oviedo et al., 2022; Karatzias et al., 2023; Johnson et al., 2022; Jenkins et al., 2025). A large body of literature suggests that social support, active stress coping, and internal locus of control are key *resiliency* factors in the face of psychological trauma (Chang et al., 2022; Cohen et al., 1985; Kaniasty, 2020; Kaniasty and Norris, 2004; Littleton et al., 2022; Rizzi et al., 2022). For example, Ukrainians have reported that staying in close proximity to, and spending time with, loved ones and family is the most commonly employed, effective coping strategy (Oviedo et al., 2022). In fact, a high quality and quantity of interpersonal relationships can serve as a buffer against loneliness, isolation, and somatic/physical ailments; the latter factors being associated with higher prevalence of adverse posttraumatic reactions (Clark et al., 2018). In the instances in which resilience is achievable in Ukraine, it is often followed by lower psychological and emotional deterioration and fewer symptoms of PTSD (Kurapov et al., 2023; Kurapov et al., 2022).

Resilience in the Ukrainian population is directly associated with coping strategies, or cognitive and behavioral means of managing internal and external demands of a traumatic or stressful event (Folkman and Moskowitz, 2004). In general, coping strategies have been defined as adaptive (which includes emotion- and problem-focused efforts) or maladaptive (which includes avoidance of stressor-induced problems and emotions) (Carver et al., 1989). Not surprisingly, Ukrainians that employ avoidant coping strategies such as withdrawal, social isolation, “giving up,” and substance abuse report higher levels of trauma (Dlugosz, 2023) and lower levels of resilience (Kurapov et al., 2022). Consistent with this, Wang et al. (2024) reported maladaptive coping to be positively associated with depressive disorder, anxiety disorders, complex PTSD [i.e., PTSD symptoms plus emotional dysregulation, negative self-concept, and relationship difficulties; (Cloitre et al., 2005)] and “traditional” PTSD, and loneliness in Ukrainian adults sampled in a 2023 study. Conversely, the latter population demonstrated a significant positive association between adaptive coping strategies and ratings of quality of life.

When considering factors that promote resiliency in the aftermath (or in the case of Ukraine *during* trauma exposure), one must account for the role of the family architecture and dynamics. For example, in Ukraine, there are traditional patriarchal family values (Ketelaars, 2019), a focus placed on the family unit, and a firm sense of collective unity; factors that one might consider to be beneficial in the face of war trauma (Gumeniuk et al., 2021). Recent studies, however, suggest that social support is more complex than simply a form of buffering against adverse mental health outcomes – to the degree that social support may be antithetical to developing and maintaining resilience. For instance, within tight knit groups of family or friends, the mutual sharing of traumatic war experiences may hamper efforts at resilience; this latter effect is described as a *war stress sharing deterioration effect* (Palace et al., 2022). It is plausible that such a deterioration effect might be worsened due to martial law imposed by the Ukrainian government in February of 2022. Under this martial law, men aged 23–60 were prohibited from leaving Ukraine without specific legal exemptions (e.g., having three or more children at home; severe disability; medical exemption) such that men aged 25–60 were made available for active duty (if already a military service member) or conscription (if civilian at the time of the invasion). Men aged 23–25 were placed on reserve lists with travel restrictions in place (U. S. D. S, 2022; EU, 2026). A layer of complexity is added to these social stressors when traumatized individuals are also exposed to real-time events or media portrayals of the ongoing violence and combat operations.

When comparing Ukrainian civilians, in general, to active military combatants, depression and anxiety symptoms were higher in civilians while PTSD symptom severity did not differ between the two groups (Ben-Ezra et al., 2023). Xu et al. (2023) identified four risk factors that may account for the higher PTSD symptoms in civilians: gender, having an urban residence, the presence of children or elderly individuals in the home, and living in a Russian-occupied area. Regarding civilian risk, it has been well established that women are at greater risk for developing PTSD after trauma than men and that PTSD is highly co-morbid with alcohol- and substance use disorders (Kessler et al., 1995; Towers et al., 2026). As such, it is apparent that civilian Ukrainian women may be at greater risk because of a confluence of factors that increase their vulnerability including exposure to war stress, alcohol dependence, and the presence of depression symptoms; a clinical presentation that has been observed in other nations facing Russian hostilities in the post-Soviet era (Ivanova and Giannouli, 2017).

## 3 Effects on refugees and the internally displaced

As alluded to above, direct exposure, witnessing, or learning about military actions leads to higher ratings of fear and anxiety in traumatized civilians (Kurapov et al., 2023). When examining trauma-exposed samples in Ukraine, it has become evident that some subgroups are potentially at greater risk. For example, individuals that were displaced from their homes within Ukraine (IDPs) or who were refugees moved outside of Ukraine have shown increased probability for developing PTSD versus those who remained in their homes (termed urban dwellers)(Johnson et al., 2022, Ben-Ezra et al., 2023, Lushchak et al., 2024). One estimate of Ukrainian refugees in Poland surveyed by Dlugosz (2023) suggests that up to 73% of the study sample reported symptoms of depression, anxiety disorders, and PTSD with 66% reporting some form of psychological distress.

As of this writing, the number of Ukrainians displaced from their homes has eclipsed 10 million with approximately 3.7 million being IDP and 6.5 million seeking asylum in other countries as refugees (UNHCR, 2024). Key factors in the greater PTSD risk in IDPs and refugees are the duration of the displacement trip, the number of days spent at a transit center, the loss of access to logistical, economic, and financial resources, as well as potential maladaptive (avoidant versus active) coping resources following trauma (Hobfoll et al., 2020; Rizzi et al., 2022). Refugees, as compared to IDPs, can have their own unique sources of risk including the migration trip itself, language barriers, socioeconomic hardship, unemployment, unstable housing, sleep disruption, and isolation due to distal separation from family and resources (Kurapov et al., 2023; Hynie, 2018; Zablocka-Zytka and Lavdas, 2023). Additionally, Ukrainian refugees tend to be largely women as men of military fighting age are restricted from traveling outside of Ukraine (Kohlenberger et al., 2023). As such, IDPs tend to move as a family with intact social support mechanisms whereas refugees are often at greater risk due to the fractured nature of their departure (Lauren and Dumont, 2023; Chudzicka-Czupala et al., 2023; Dlugosz, 2023). It is noteworthy, however, that some previous work has revealed no differences between IDPs and refugees with respect to PTSD symptoms, markers of resilience, nor employment of avoidant coping strategies (Khailenko and Bacon, 2024).

The fractured nature of refugees’ departure can include leaving family members behind due to conscription and military service for male family members (typically aged 23–60) (U. S. D. S, 2022), older age, health problems, or a necessary homeland occupation (e.g., physician) and this, in turn, can lead to a sense of “survivor syndrome” in those able to leave (Grauer, 1969). This sense of “survivor syndrome” may be exacerbated in those refugees who experienced the death of friends or loved ones prior to their departure. The chaotic nature of leaving may prevent refugees from locating the remains of those they lost, having proper funerals, or saying goodbye and obtaining closure (Zablocka-Zytka and Lavdas, 2023); all of which can lead to complicated grief that, in turn, can increase symptoms of anxiety, depression, and psychological stress (Hutul et al., 2026).

One widely employed method of assessing the degree of risk and resiliency for posttraumatic stress reactions in displaced Ukrainians has been comparison studies between those displaced and a sample of the native citizenry of their new host country. For example, Poland has received approximately 1 to 1.6 million Ukrainian refugees as of early 2023 (Statystyczny, 2024; Rizzi et al., 2023). Jaworski et al. (2025) reported that Ukrainian refugees exhibited greater psychological distress than Polish citizens, in general, as well as stronger social networks, most notably with extended family (Jaworski et al., 2025). Multiple regression analyses in the Jaworski study identified refugee status, increased loneliness, younger age, and psychiatric illness history as predictors of greater psychological distress. It is notable that 75% of the refugee study sample identified a clinically relevant degree of psychological distress; a finding that is consistent with prevalence of psychological distress in Ukrainian refugees transported to Germany (Buchcik et al., 2023). Further, when compared to the general population of Taiwan and Poland, Ukrainian refugees exhibit higher scores on measures of anxiety, depression, and posttraumatic stress (Chudzicka-Czupala et al., 2023). Setting aside the obvious previous and ongoing exposure to Russian hostilities in their home country, Ukrainian refugees exhibit greater psychological distress as compared to members of a host society (e.g., Poland) due to: (1) unreliable access to Ukrainian-based monetary funds from the host country, (2) unstable living conditions in temporary shelters, host family residences, or rental properties, and (3) restriction of rights, segregation, and discrimination from the host society (Zablocka-Zytka and Lavdas, 2023).

While Poland serves as a representative example of the dynamics between a host-country and the refugees that it receives, there are several European countries that currently serve as hosts for displaced Ukrainians. In fact, cross-national differences in host/refugee circumstances, including psychological distress levels, resiliency markers, and coping strategies, were very recently examined by Szkup et al. (2026). In the Szkup study, Ukrainain refugees displaced to Poland, Germany, Spain, Cyprus (member of the EU), Slovenia, and Serbia were surveyed online (Szkup et al., 2026). Psychological distress levels were highest in refugees living in Slovenia, Portugal, Spain, and Germany and were lowest in Cyprus and Poland. The most resilient Ukrainian refugees were found in Poland and Germany while the least resilient lived in Spain. Refugees in Slovenia, Poland, and Germany reported the greatest use of adaptive coping measures while those living in Cyprus and Serbia indicated decreased use of adaptive coping strategies. The authors attribute these cross-national differences to institutional, social, and policy practices that include elements such as access to services, stability and success of refugee reception programs, and the degree of support from host governments and residents in the area of sociocultural integration; all factors that can determine whether or not coping strategies can be successfully employed.

A recent review by Rizzi et al. (2023) identified five primary categories of coping strategies employed by Ukrainian refugees and internally displaced individuals: (1) emotion-focused, (2) problem-focused, (3) avoidance, (4) faith-based, and (5) those fostering a greater sense of belonging(Rizzi et al., 2023). Interestingly, Khraban (2022) reported a progression from one coping strategy to another in Ukrainian civilians during the initial phases of the war. Non-adaptive, emotionally-focused coping strategies were widely employed during the first 5 days of the conflict which were then followed by problem-oriented strategies from days 6 through 10. Next, Ukrainians exhibited strategies focused on belongingness, hope and faith from days 11–15. The wide ranging use of emotion-, problem-, and avoidance-focused coping strategies was also seen in a largely non-displaced Ukrainian civilian sample assessed during the first month of the war (Xu et al., 2023). It appears that as the Russian hostilities continued in the coming months that an intact social network, maintenance of communication with family and loved ones, and faith emerged as leading coping strategies employed by displaced Ukrainians (Oviedo et al., 2022; Rizzi et al., 2022).

## 4 Effects on urban dwellers (non-displaced)

While it has been clearly established that displaced individuals are at higher risk for posttraumatic psychological distress, one must also consider the urban dwelling population that remained in their primary area of residence in Ukraine. As expected, reports from early in the conflict have estimated that close to half of the general Ukrainian population has experienced symptoms of psychological distress, anxiety, and depression during the 2022 Russo-Ukrainian war (Xu et al., 2023). As described by Xu et al. (2023), the population sampled in their study was drawn from the entire territory of Ukraine, all respondents had been exposed to warzones at some point in their lifetimes, but the majority were not in an area currently occupied by Russian forces (96.4%) nor were there active combat operations in their geographical area (93.5%). Additionally, 90% of the respondents in the Xu study reported being currently employed or as students and they participated by completing mobile device-based surveys and questionnaires. These factors strongly suggest that the sample was comprised of non-displaced urban dwellers but this is not explicitly stated by the authors.

A recent study of Ukrainian civilians exposed to war-related trauma identified prior history of psychopathology, personality features, and sociodemographic factors as primary influences for one’s risk versus resilience for PTSD and distress symptoms (Ho et al., 2023). At the time of their participation, respondents in the Ho study were part of the Mental Health of Parents and Children in Ukraine Study, largely resided in an urban area (75.0%), were married or living with their partner (78.0%), completed an undergraduate degree (62.7%), and more than half of the sample (59.4%) was employed full-time or part-time; all factors suggesting that these were mostly non-displaced urban dwellers at the time of their participation although this is not explicitly stated.

An interesting risk versus resilience factor for the development of posttraumatic responses in the Ukrainian, non-displaced population is the role that *perceived support from the West* which would include military aid and support collectively from NATO, or individually from the European Union, the United Kingdom, or the United States. Palace et al. (2022) reported that perceived, or expected, support from the West was associated with higher anxiety in young adult Ukrainian students who were not displaced. Specifically, these students felt that involvement of the West (and potentially large losses in the Russian military) might escalate the conflict toward the use of weapons of mass destruction (Palace et al., 2022). The work by Palace et al. (2022) also highlights the important role of including student populations when assessing the psychological consequences of an active warzone within non-military, civilian populations (Chabake et al., 2025).

The use of drone warfare has become much more prevalent in the more recent global conflicts including the 2022 Russo-Ukrainian War. For example, Russia has frequently employed drones to attack Ukrainian military and civilian targets and, as such, some have termed the ongoing Russo-Ukrainian war to be the first “drone war (Cropsey, 2024).” The use of this type of unmanned aerial vehicle for attacks against civilian and military targets has the effect of both terrorizing and demoralizing the local populations (Meheut, 2023); effects that represent significant barriers to achieving resiliency.

## 5 Effects on host country

The psychological consequences of the Russo-Ukrainian war are not limited to Ukrainians, both non-displaced and resettled, but can also extend to the population of the host country receiving refugees. Providing support to refugees for an extended period of time can lead to burnout, feelings of helplessness or hopelessness, depression, exposure to secondary trauma, reluctance to continue aid, and the potential for hostile feelings toward the refugee population (Zablocka-Zytka and Lavdas, 2023; Messiah et al., 2016). Exposure to secondary trauma can occur if hosts are repeatedly listening to recollections of war, loss, and displacement, which can then, in turn, increase mood and anxiety-related symptoms for the hosts (Duray-Parmentier, 2022). Additionally, conflicts between hosts and refugees may arise due to boundary issues and compassion fatigue and as personal boundaries are missing or become eroded over time while the two groups share living spaces (Banulescu-Bogdan et al., 2024). Simple logistical issues such as maintaining different daily routines can lead to a cascade of adverse outcomes up to and including cessation of the arrangement. Further, psychological strain can build for hosts and refugees because of feelings of guilt and mounting financial pressures as many hosts experience anxiety over their guests’ uncertain futures and rising costs of living, most notably as the end of housing commitments approach (Seguin et al., 2023).

Complex psychological reactions on the part of refugees and their hosts may be especially true for homestay situations in which permanent citizens of a host country provide temporary shelter in their own residences or on their property for refugees in an effort to facilitate shelter, safety, and basic needs (Bassoli and Campomori, 2022). This typically occurs as a transition until refugees establish independence and long-term stability. On the positive side, homestay situations can be a source of a new, family-like environment in which resources are shared, can aid refugees with integration into the local society, and may foster a sense of autonomy and independence for displaced individuals (Bassoli and Luccioni, 2023; Burrell, 2024; Czischke and Huisman, 2018; Mahieu and Van Caudenberg, 2020). On the negative side, hosts may begin to harbor feelings of anger, frustration, and resentment if the temporary housing situation becomes prolonged (Zablocka-Zytka and Lavdas, 2023; Messiah et al., 2016). Relatedly, resettled refugees may experience similar feelings if their goals of becoming more independent and permanently re-housed are repeatedly thwarted (Zablocka-Zytka and Lavdas, 2023; Messiah et al., 2016). Importantly, the homestay environment can have a significant impact on refugees’ physical and mental health as these circumstances involve access to sociocultural integration opportunities, psychological support networks, and primary healthcare (Al-Saqaff, 2024).

In their recent review, Al-Hamad et al. (2024) identified six primary areas regarding motivations to, and barriers hampering, host/refugee homestay situations which are: cultural exchange, empathy and compassion, humanitarian concern, structured programs, moral awakening, and social innovation. These areas exist on a conceptual continuum in which a higher degree of each of these is associated with better outcomes for refugees (e.g., assimilation and adaptation for refugees) and their hosts with lower degrees being linked to poorer circumstances (e.g., increased psychological distress in both hosts and refugees) (Al-Hamad et al., 2024). A similar continuum is reported in the Al-Hamad review with respect to key factors that contribute to the success or failure of host-refugee relationships: empathy and communication, cultural proximity (i.e., similarity between cultures), gender dynamics/roles, sociopolitical context (e.g., governmental and public views toward refugees), household dynamics, financial constraints, duration of stay, use of technology, intermediaries, and radical cosmopolitanism (i.e., a focus on mutualism, engagement, and equality). Areas of focus such as those listed above have the potential to improve the host/refugee relationship such that each party emerges with broader sense of psychological health and well-being. Intervention strategies and mitigation steps for achieving such goals will be further discussed below.

## 6 Future directions for intervention and mitigation

**Figure 1:**
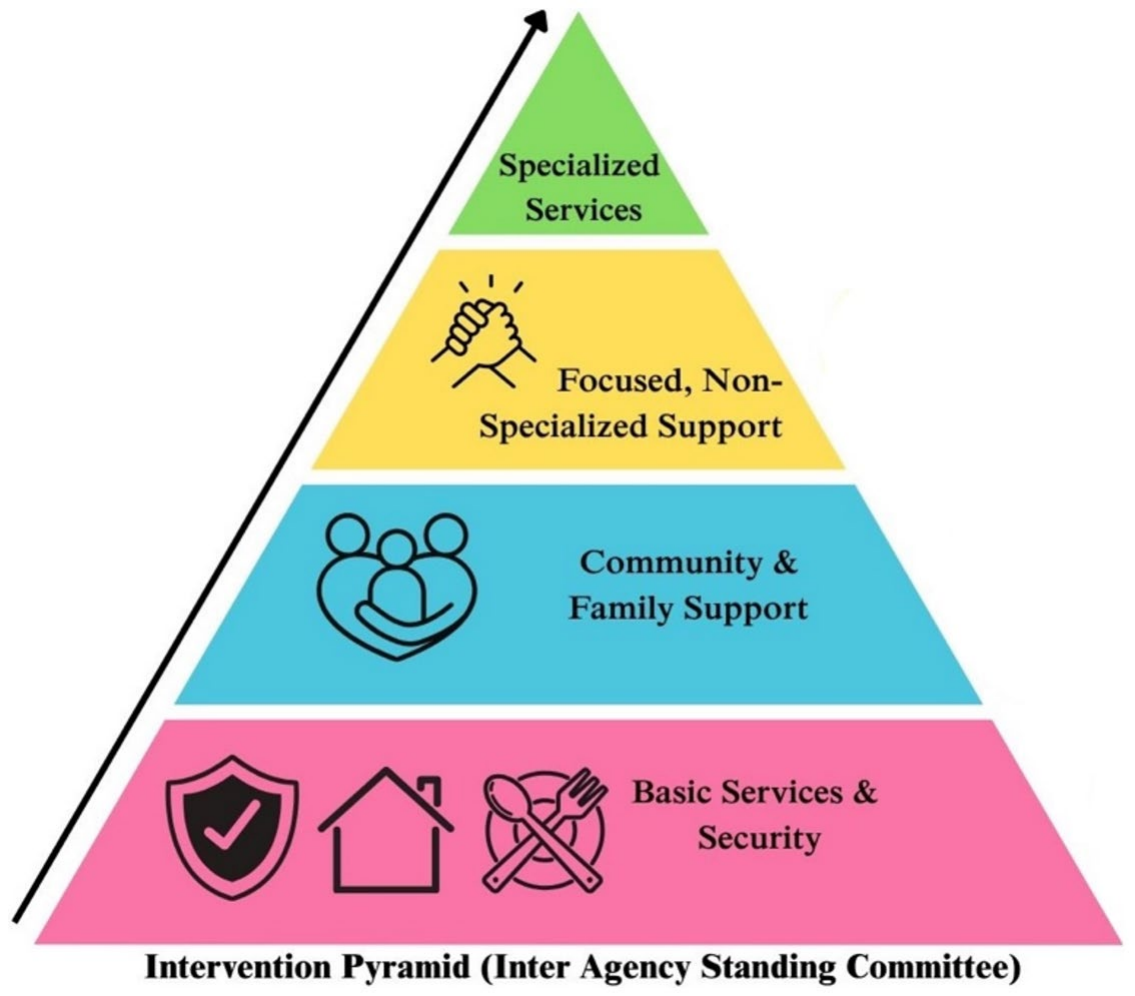
In this schematic, the intervention pyramid schema proposed by the Inter-Agency Standing Committee (IASC), which highlights a need to secure basic survival needs and security prior to advancement to the higher levels focused on community, family, and self IASC, 2021.

There is a clear need for interventions that target PTSD symptoms, build resilience, and foster more adaptive coping strategies in refugee and displaced populations in Ukraine and within other global theaters of conflict. In order to accomplish the latter, it is critical to establish systematic mental health monitoring of these traumatized populations with an emphasis on quality of life outcomes (Wang et al., 2024). This monitoring may include licensed, trained professionals but may also require recruitment and training in basic mental health first-aid for non-medical professionals (e.g., case workers, transport officials) who are more likely to interact with refugee populations (Javanbakht, 2022) or through programmatic self-report (see Figure 1 for the United Nations’ Inter-Agency Standing Committee recommendations for monitoring and intervention). As an example, a small study of adolescent Ukrainian refugees by Catani et al. (2023) supported the feasibility of deploying classroom-based mental health screening and the authors detected a high degree of PTSD symptoms in this sample.

Bolstering the resources available to those displaced through stable shelter and financial support are likely to have secondary positive effects on mental health and quality of life. At the psychological level, resources that promote adaptive coping strategies such as psychoeducation, addiction recovery, and cognitive behavioral therapy (CBT) may allow individuals to shift away from maladaptive strategies. Examples of potential adaptive coping strategies include, but are not limited to, art, music, humor, religion, acceptance, social support (peer, familial, pets), planning, and positive reframing (Kim et al., 2023; Gever et al., 2023; Miliutina et al., 2023). It should be noted here that there is much empirical work to be done regarding the effectiveness of the aforementioned strategies.

Interventions to foster improved well-being and resilience in displaced populations, such as the Ukrainians alluded to throughout this paper, may be most effective if they target social capital, or the social resources available to an individual through interpersonal ties to others and communities (Rouxel et al., 2015; Johnson-Singh et al., 2024). Social resources can include both subjective (e.g., trust, belonging, mutual reciprocity) and objective (e.g., social networks, shared norms, collective experiences) features. Social capital is also characterized by the proximal/distal nature of connections such that ties might include bonding (i.e., close associations with co-ethnic peers and family), bridging (i.e., ties with culturally diverse groups), or linking [i.e., vertical associations with institutions and power structures (Johnson-Singh et al., 2024)]. Higher connectedness along these ties is associated with decreased depression, anxiety, and posttraumatic stress symptoms in refugees; most notably when these connections promote a sense of trust, security, and social belonging (Rouxel et al., 2015; Johnson-Singh et al., 2024; Ehsan et al., 2019). Targeting bonding capital can buffer against loneliness and isolation while instilling feelings of identity and safety and this may comprise informal (i.e., familial) or formal (i.e., co-ethnic gatherings) means. An interventional focus on horizontal bridging and vertical linking capital may increase access to community/public resources, repair broken institutional trust, or eliminate barriers to care and integration. It is worth noting that the latter requires significant “buy-in” from the host country’s citizenry as well as national and local governmental bodies (Villalonga-Olives et al., 2022; Habib et al., 2020). This appears to be the case for Ukrainian refugees in Poland where approximately 80% of the population supported free access to healthcare and education for refugees (Babicki et al., 2023). However, other host nations, such as Germany, the United Kingdom, and the Netherlands, have a more polarized approach to refugee populations with national security concerns on one end and humanitarian empathy on the other (Al-Saqaff, 2024; Sirriyeh, 2013; Baban and Rygiel, 2017).

The development of systematic and scalable intervention and mitigation protocols should also address cultural, spiritual, and social circumstances. For example, Skalski-Bednarz et al. (2024) reported that exposure to war-related activities such as bombings and missile attacks was associated with less forgiveness, a reduced sense of faith, and elevated anger toward God in Ukrainian refugees; consequences that likely impair resiliency efforts and traumatic stress management. With regard to social contexts, intervention and mitigation efforts should also include limiting engagement with media and social media coverage of the ongoing war as this added exposure can worsen war-related psychological distress (Rozanov et al., 2019).

The Inter-Agency Standing Committee (IASC), the highest-level humanitarian coordination forum within the United Nations system, has proposed a four-level pyramid schema for organizing mental health support for refugees specifically (See Figure 1) (IASC, 2021). This pyramid model requires efforts from the host country’s society in general, international organizations, non-profit citizen-based groups, as well as specialized (e.g., clinical) and non-specialized community members. Level 1, at the base of the pyramid, includes provision of basic services and security (physical, emotional, and informational). Physical security encompasses a place to live, medical care and medication, food, and hygiene products (Murphy et al., 2022). Emotional security has been alluded to above and consists of connecting with family, friends, neighbors, and fellow refugees (Rouxel et al., 2015, Johnson-Singh et al., 2024, Ehsan et al., 2019). Informational security is focused on providing refugees with reliable communications regarding the refugee transition process as well as updates about the ongoing situation in their homeland. The base of the pyramid also includes access to educational resources such as health education, stress management, and establishing means of successfully integrating a refugee’s own Ukrainian culture with that of the host country. An app called EmotionalAid^1^ is one example of an educational resource available to refugees that has been deployed in Poland (Zablocka-Zytka and Lavdas, 2023). A final element of the pyramid’s base is psychological first-aid (PFA), referenced above, which has been developed in coordination with the World Health Organization, the War Trauma Foundation, and World Vision International (World Health Organization, War Trauma Foundation & World Vision International, 2011). PFA is conceptualized as humane, supportive responses to a fellow human being who is suffering and who may need support, most notably after a traumatic event or emergency situation^2^.

The second level of the IASC pyramid focuses on strengthening community and family supports which can include social-network based interactions, child-friendly spaces, and community-based traditional support. First, an emphasis on social-network interactions ensures that refugees can maintain life activities consistent with their homeland culture and beliefs in the presence of fellow displaced refugees. Second, the creation of child-friendly spaces is part of an umbrella effort to support refugees who are parents in managing their own stress and that of their children. Third, community-based traditional support systems include the involvement of non-government organizations that can provide training in areas such as coping with extreme emotional states, the basics of effective communication, protection against further violence, and positive psychology techniques. Community-based efforts may also target assistance with educating school-age refugees as well as with easing the transition into the new country’s language, employment, cultural, and social systems. This transition may be most effective if there is a reciprocal exchanging of cultural ideals and beliefs between the refugee population and the new country of residence’s people.

The IASC’s third level of the pyramid places its emphasis on the role of non-specialized supports and personnel. This level may include person-to-person (P2P) interactions between primary health care (and not mental health) professionals and refugees and P2P interactions between community workers (e.g., social workers, teachers, clergy members) and refugees in the area of psychosocial and emotional support. Note that this is a degree of interaction that exceeds that of PFA and is much more comprehensive.

The top of the IASC pyramid for organizing mental health support for refugees places its focus on specialized services including obtaining mental health care from mental health specialists such as psychiatrists, clinical psychologists, and trained psychotherapists. Rather than addressing general stress management and coping skills, which are part of the lower pyramid levels, level four includes treatment for clinically significant psychological distress up to and including psychiatric disorders such as PTSD; this may also include the treatment of psychiatric problems that existed prior to war-related trauma exposure. A critical element of achieving the goals of level four is the creation and maintenance of centers of care for refugees and this objective may be thwarted by infrastructure and financial limitations of the host country.

It is important to note that the implementation of any prevention or mitigation strategies for traumatized populations such as that in Ukraine requires at least three important elements. First, it requires an openness to intervention by the refugee population and this is not always the case. For example, more than half of the Ukrainian participants surveyed by Chudzicka-Czupala et al. (2023) reported that they would not seek psychological help despite having a significantly higher prevalence of war-related mental health consequences. Second, as an extension of the first element, is a willingness of refugees themselves to be involved in providing the care and support delineated in the above pyramid to their fellow displaced countrymen (Noha et al., 2022). Third, prevention and mitigation strategies should be based on the experiences of other countries that have taken in substantial refugee populations (e.g., Germany, Israel, Greece).

## 7 Conclusion

The psychological impact of the 2022 Russo-Ukrainian War is likely to be widespread and long-lasting. The ongoing war, and the subsequent post-war era, will continue to be characterized by symptoms of anxiety, depression, substance abuse, and PTSD within home country residents in the line of fire (or involved in post-war recovery efforts), individuals displaced within their home country, refugees living in new host countries, and citizens of the nations that receive refugees. Yet, there are signs of promise emerging from the ongoing empirical study of these groups such that intervention strategies have been largely conceptualized and, in some cases, installed within certain limited areas. The success of these interventions will depend on a strong sense of motivation and willingness to both offer and receive help; efforts that can be bolstered by factors including successful cultural integration and assimilation, robust levels of empathy and compassion, shared humanitarian concern, increased and improved structured programs, higher moral awakening and awareness, and a focus on social innovation. There are valuable lessons to be learned from observing the Ukrainian response to Russian hostilities that may be further applied to war-torn regions including the ongoing Syrian Refugee Crisis and mass displacement of Palestinians in Gaza.

## Footnotes

1
https://emotionalaid.com/home/toolkit

2 Please see https://iris.who.int/server/api/core/bitstreams/e7e129fb-b306-496d-84a5-67bb70abc130/content for a detailed description.
